# Effects of ayahuasca in preclinical studies with animals: a systematic review

**DOI:** 10.1590/1414-431X2025e14687

**Published:** 2025-08-29

**Authors:** A. Walsh-Monteiro, S. Morato, F.A.R. Uribe, A. Gouveia, J.S. Pedroso

**Affiliations:** 1 Laboratório de Neuroquímica e Comportamento, Coordenação de Ciências Biológicas, Campus Tucuruí, Instituto Federal do Pará, Tucuruí, PA, Brasil; 2 Laboratório de Comportamento Exploratório, Faculdade de Filosofia, Ciências e Letras, Universidade de São Paulo, Ribeirão Preto, SP, Brasil; 3 Laboratório de Desenvolvimento e Saúde, Instituto de Filosofia e Ciências Humanas, Universidade Federal do Pará, Belém, PA, Brasil; 4 Laboratório de Neurociências e Comportamento, Núcleo de Teoria e Pesquisa do Comportamento, Universidade Federal do Pará, Belém, PA, Brasil

**Keywords:** N,N-Dimethyltryptamine, Hallucinogens, Serotonin receptor agonist, Monoamine oxidase inhibitors, Animal models

## Abstract

This systematic review investigates the effects of the ayahuasca beverage (Aya) in various animal models. Using the PRISMA protocol and adhering to the Cochrane Handbook for Systematic Reviews, a comprehensive selection of 2,359 documents was identified from the Web of Science, Medline, and Scopus databases between 2012 and 2022. Following the inclusion and exclusion criteria, 14 articles were included in the final analysis. The analysis revealed a diversity in the selection of animal models that included different developmental stages and various forms of Aya intoxication, including acute and chronic doses, and varying concentrations of the active principles. The studies revealed that Aya causes significant alterations in the motor and cognitive behavior of animals, especially associated with the serotonergic system, which seems to contribute to the negative symptoms also observed in schizophrenia and depression. Despite the evidence found, this review highlights the scarcity of more robust pre-clinical studies with methodological standardization to make more conclusive comparisons, especially given the need to identify potential toxic and neurochemical effects of Aya on organisms for a safer assessment of its therapeutic use.

## Introduction

**Figure 1:**
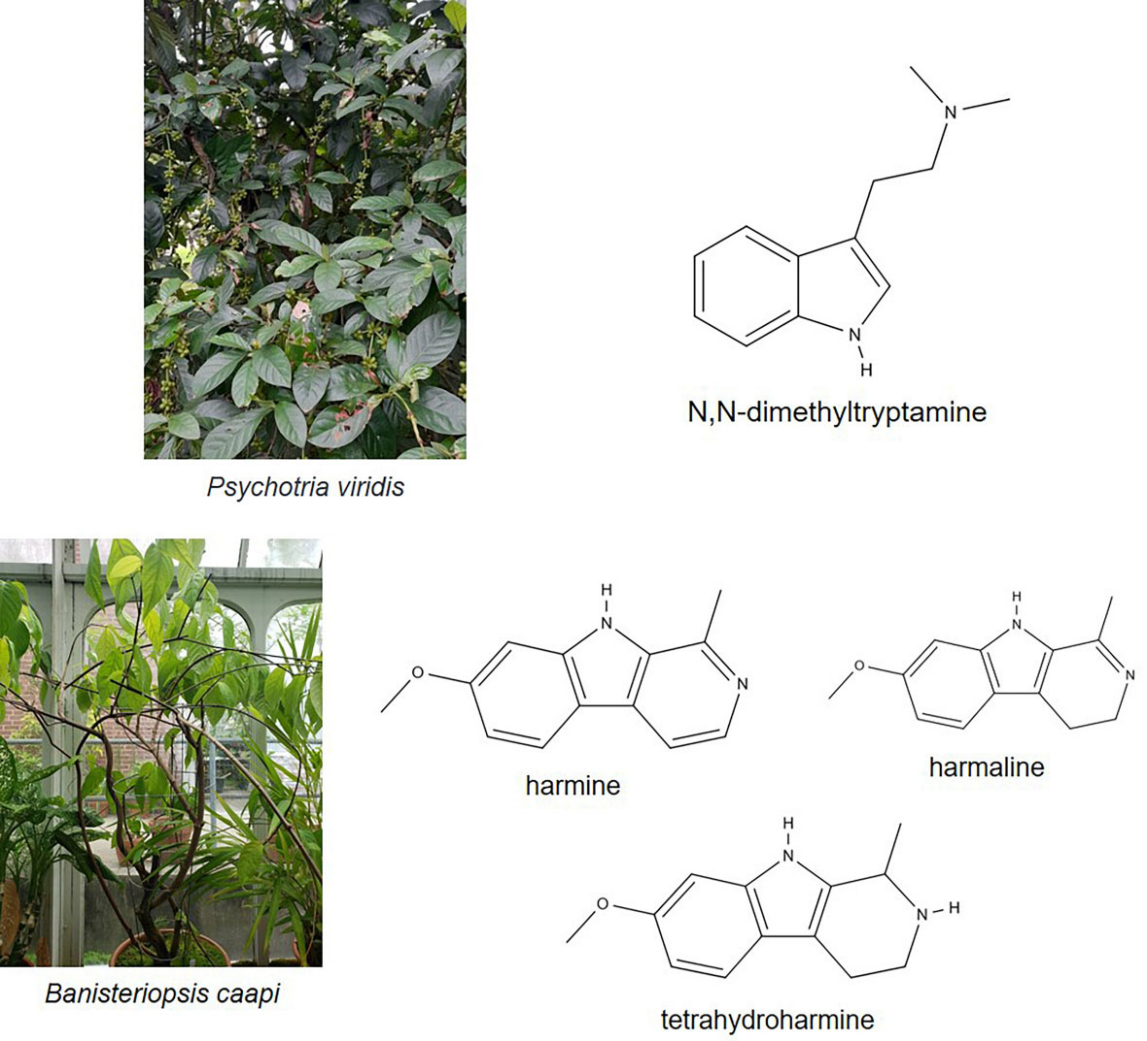
Four main alkaloids in ayahuasca beverage and photographs of the plants. N,N-dimethyltryptamine found in *Psychotria viridis*, and harmine, harmaline, and tetrahydroharmine found in *Banisteriopsis caapi*. Chemical structures extracted from https://molview.org/ and images of the plants found at https://identify.plantnet.org/.

Ayahuasca (Aya) is a psychoactive beverage that is composed of a combination of two plants: *Psychotria viridis* Ruiz & Pav. (Rubiaceae) and *Banisteriopsis caapi* (Spruce ex Griseb.) C.V. Morton (Malpighiaceae) (Figure 1). It is traditionally used for medicinal and ritualistic purposes by local indigenous and mestizo communities in the Amazon region (1). In Brazil, the ritualistic use of Aya was regulated by Resolution No. 1 of January 25, 2010 by the National Council for Drug Policies, which defines standards for cultivation, preparation, transportation, and religious use (2). In the context of traditional medicine, Aya has been used to treat symptoms related to mental health, including mood disorders, neurodegeneration, and substance dependence (3-5). Its active compounds include N,N-dimethyltryptamine (DMT), which is extracted from the leaves of *P. viridis*, and three β-carbonyl derivatives (harmine, harmaline, and tetrahydroharmine (TTH)), which are extracted from the vine of *B. caapi* (6-8) (Figure 1). DMT, the component that induces Aya's hallucinogenic symptoms, is an alkaloid that is abundant in nature (9) and has even been identified in mammals (10). It is an agonist of serotonin (5-HT) receptors, predominantly 5-HT2A, which induces a hallucinogenic effect on the central nervous system (11,12). In relation to β-carbonyl alkaloids, harmine and harmaline have been identified as inhibitors of the enzyme monoamine oxidase A (iMAO-A), while TTH has been characterized as a weak inhibitor of 5-HT receptors (13,14).

Of the components of Aya, DMT is the most studied and of greatest pharmacological interest (15,16). It belongs to the group of indolylalkylamines of the tryptamine type (17), with a total or partial agonist effect on 5-HT2A in the central nervous system (11,12). The psychotropic effects of DMT include euphoria, visual hallucinations, and speech disorders (15,16).

Structurally, DMT is analogous to 5-HT, melatonin, and certain vasoconstrictors (18,19). These characteristics allow molecular manipulation to create synthetic products that retain psychedelic properties or eliminate these hallucinogenic characteristics and focus on other therapeutic potentials (17,18).

The similarity of DMT to 5-HT is associated with the substance's ability to bind to different serotonin receptors such as 1A, 1B, 1D, 2A, 2B, 2C, 6, and 7 (18,20,21). However, studies suggest that the hallucinogenic effects are relatively concentrated in 5-HT2, which renders this group of receptors interesting pharmacological targets for study (22,23). It is imperative to emphasize that the primary focus is on 5-HT2A, which is more extensively distributed in the cerebral cortex of mammals than the other receptors (24), and has also been identified in the striatum, hippocampus, and amygdala (25).

A number of significant positive effects have been reported by users of Aya beverage, including the alleviation of depressive symptoms. This phenomenon appears to be associated with the long-term effects of DMT exposure, which has been observed to potentiate the expression of genes that encode regulatory factors, such as *c-fos*. These regulatory factors have been linked to synaptic plasticity, memory, attention, and personality (26-30).

Conversely, in sufficient concentrations, β-carbolines have the capacity to induce physiological and psychological effects on users by modulating the effects of neurotransmitter amines or specific receptors (4,31). It has been demonstrated that harmine and harmaline exert an impact on the release of dopamine, thereby influencing the pathways of this neurotransmitter (4,32). Harmine has been observed to inhibit both the dopamine transporter (DAT) (33) and tyrosine-phosphorylation-regulated kinase 1A (DYRK1A), which is responsible for modulating DAT membrane traffic. In both cases, the inhibition of these processes has been shown to contribute to an increase in dopamine levels within the synaptic cleft (32).

Following the ingestion of Aya, the neuropsychological effects become apparent within approximately 40 min, inducing alterations in the state of consciousness, including the intensification of mental images and affective regulation (34-36). The underlying mechanism of these effects is attributed to the combination of DMT with β-carboline alkaloids, which have been shown to inhibit the degradation of DMT by MAO-A, thereby enhancing the effects on serotonin receptors (12,34,37).

A comprehensive review of the literature on the use of Aya in human subjects reveals a myriad of purported benefits, including the promotion of neuroplasticity and neurogenesis and an augmented release of brain-derived neurotrophic factor (BDNF). In addition, improvements in attention and a positive emotional state have been reported (38-41). Nevertheless, there are still knowledge gaps concerning the mechanism of action, effects, and risks of Aya use, particularly in preclinical studies. As with any substance with potential therapeutic use, rigorous testing is required to assess both the efficacy and safety of Aya use. In this context, preclinical studies with animal models and *in vitro* tests are imperative for preliminary evaluation. The present study has organized a systematic review of the behavioral, biochemical, and physiological effects of Aya components in preclinical animal models.

## Methods

### Study characterization

This systematic review was conducted in accordance with the PRISMA (Preferred Reporting Items for Systematic Reviews and Meta-Analyses) protocol (42), ensuring the rigor and transparency of the review process. The study also followed the guidelines set out in the Cochrane Handbook for Systematic Reviews (43). The review protocol was submitted and approved by the International Prospective Registry of Systematic Reviews (PROSPERO) under the registration number CRD42022333011.

### Database search

The descriptors were selected from MeSH (Medical Subject Headings), adopting English as the only language. The descriptors selected were: “ayahuasca”, “AYA”, “animal model”, “n,n-dimethyltryptamine”, “banisteriopsis”, “harmaline”, “harmalan”, and “DMT”. The Boolean operator AND was employed between descriptors, with all possible combinations (two descriptors at a time) being considered. The databases searched were Web of Science, Medline, and Scopus, and the publication period was between January 2012 and December 2022.

### Study selection strategy

The management of search results was conducted using the Mendeley Desktop software (USA, version 1.19.8). Initially, duplicate studies and studies from the grey literature (i.e., abstracts, books, chapters, texts without peer review) or published in languages other than English were excluded. The final inclusion criteria were as follows: experimental studies with non-human animal models published in English between 2012 and 2022 investigating the active components of ayahuasca (DMT and β-carbolines). Non-experimental studies (cross-sectional, case-control, longitudinal), involving humans or reviews, were excluded from the analysis.

The selection of articles was based on title analysis, followed by an evaluation of the abstract. In instances where the selection of articles was uncertain, the article was read in its entirety. The entire process was conducted by two independent reviewers (A.W.-M. and F.A.R.U.), and a third reviewer (A.G.Jr.) was consulted in case of disagreement, seeking consensus for the inclusion or exclusion of studies.

### Data extraction

The process of data extraction was conducted by two independent reviewers (A.W.-M. and S.M.) using a standard form from the Systematic Review Data Repository (SRDR) (44). The information included author(s) and year of publication, sample (characteristics of the species used, doses and forms of administration, tests applied, and other relevant information), primary results (main conclusions), and secondary results (measures of association).

### Methodological quality assessment

The methodological quality of the studies was assessed using the SYRCLE RoB risk of bias tool for animal studies (45). This tool systematically evaluates the potential for bias across ten distinct domains, categorizing studies into low, high, or unclear risk of bias.

### Synthesis and analysis

A narrative meta-synthesis of the results was performed, based on the evidence reported in the studies. However, conducting a meta-analysis was not feasible due to the heterogeneity of the data.

## Results

### General characteristics of included studies

**Figure 2:**
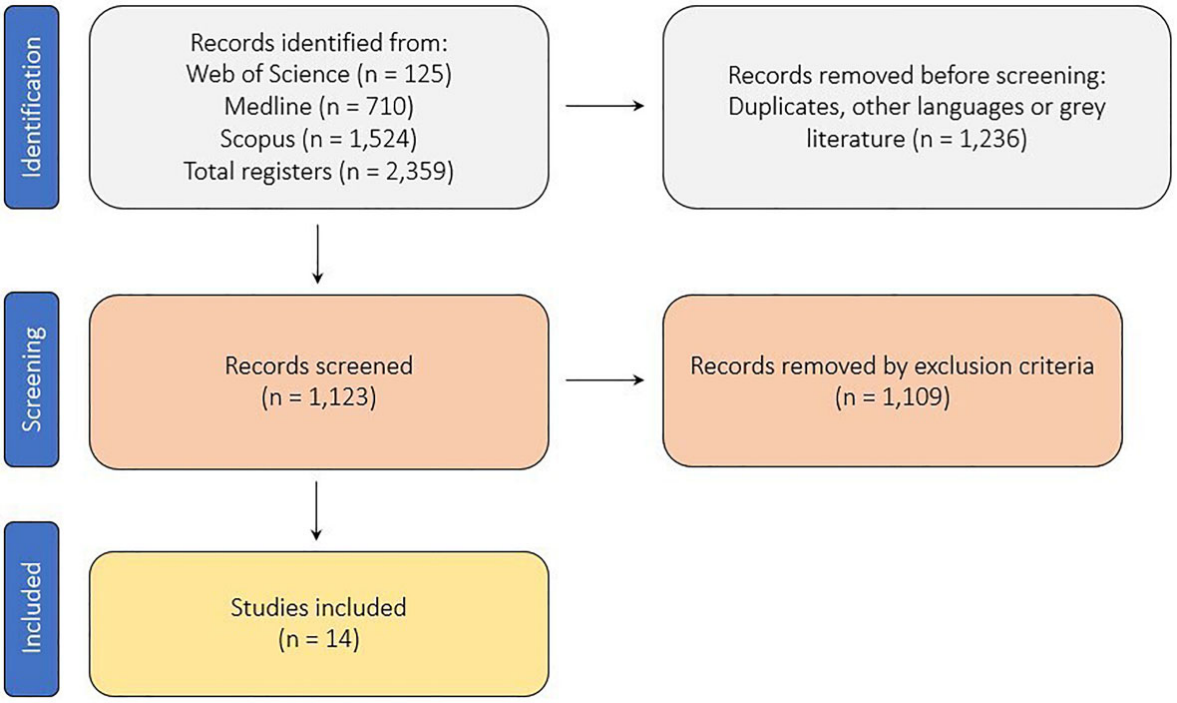
PRISMA flowchart of article selection (adapted from Page et al. 2021; doi: 10.1136/bmj.n71).

Following the completion of searches in the three databases, a total of 2,359 documents were identified. After the removal of duplicates, grey literature documents, and documents written in languages other than English, 1,123 articles remained for screening according to the inclusion criteria. After the evaluation of the inclusion and exclusion criteria by the two reviewers, 14 articles fully met the criteria of the present study. Figure 2 shows the document selection flowchart using the PRISMA protocol.

The articles selected for inclusion in this study were conducted in Brazil and used a variety of animal models, administration schedules, and concentrations of substances. Animals were exposed to dilutions of Aya beverage or lyophilized Aya.

The species used in this study encompassed adult male (46-48) and nulliparous female Wistar rats (49), male Swiss mice (50,51), male albino mice (52), C57BL/6 of both sexes (53), male mice (54,55), adult zebrafish (56,57), and zebrafish embryos (58). Additionally, marmosets (59) were used in one study.

In addition to the diversity of species (with variations in sex, strains, and ages), different forms of administration, such as oral, intraperitoneal, and dilution in water and other means, were also adopted. The concentrations of the substance under investigation varied as did the preparations, such as per kilogram of animal or per liter of animal. Furthermore, acute, subchronic, and chronic administrations were adopted.

Table 1 presents a summary of the general data from the included articles. As all of the included studies were of an experimental nature, negative control groups were included for comparison. Furthermore, all administration of substances that was performed orally (*vo*) occurred by gavage.

**Table 1:** Characteristics and primary results of the included studies. Aya: ayahuasca.

Reference	Sample	Findings	
		Primary Outcomes	Measure of Association
Alvarenga et al. 2014 46	Male Wistar rats trained for sexual experience Doses: 250-1000 µg/mL (*vo*) Interventions: paradoxical sleep deprivation (PSD), 96 h. Tests: Arena for evaluation of sexual behaviors; biochemical analysis	Any dose of Aya in non-sleep-deprived animals impaired the sexual performance of the individuals. When combined with sleep deprivation, it had heterogeneous effects. Low doses of Aya combined with sleep deprivation showed improvement in sexual performance. Animals exposed to PSD-500 Aya had reduced progesterone levels. No differences were found in the number of mounts and ejaculations. Animals exposed to Aya did not differ in testosterone and corticosterone levels between control and PSD.	Reduction in intromissions: P<0.002, P<0.004, and P<0.006.PSD-500 µg/mL: reduction in intromissions: P<0.01 and P<0.006PSD-250 µg/mL: increase in intromissions: P<0.01 and P<0.02PSD-500 µg/mL: reduction in progesterone, P<0.03
Pic-Taylor et al. 2015 49	Nulliparous Wistar rats Doses: 15× and 30× ritual, Fluoxetine (Flx, positive control) Tests: open field (OF), elevated plus maze (EPM), forced swim (FS), c-fos activation and toxicity	Aya and Flx caused reduced locomotor activity in the OF and EPM and rearing and grooming movements compared to controls. Aya increased swimming and reduced immobility in the NF compared to controls. Aya 30 × increased activity in serotonergic neurons (DRN: dorsal raphe nucleus, amygdala, and hippocampus), causing non-permanent lesions. The lethal oral dose for Wistar rats is greater than 50× the ritual dose.	OF: Aya or Flx *vs* control (locomotion, P<0.05; rearing, P<0.01). Grooming, Aya only, P<0.05 EPM: Aya or Flx *vs* control (closed arms, P<0.05 - P<0.001; 50x open arms, P<0.05) FS: P<0.001 C-fos: P<0.05 and P<0.001
Oliveira-Lima et al. 2015 50	Male Swiss EPM-M2 mice Doses: 30-500 mg/kg (*ip*) Interventions: ethanol (Etoh) 1.8 g/kg (*ip*); sensitization and counter-sensitizationTest: OF	Aya inhibited behavior associated with Etoh dependence. Aya effectively reversed the expression of ethanol effects by inhibiting the reinstatement of Etoh-induced behavioral sensitization.	Hyperlocomotion: Aya doses reduced Etoh effect, P<0.05 Sensitization: Aya doses reduced Etoh effect, P<0.05
Correa-Netto et al. 2017a 54	Male C57BL/6 mice Dose: 1.5 mL/kg (*vo*), 2× week (4-28 doses) Groups: childhood, adolescence, adult, and intersections Tests: OF, Morris water maze (MWM), and EPM	There was no variation in locomotor activity in the OF and open arms of the EPM, between Aya and control at different ages. Aya in childhood caused an increase in risk assessment (anxiety) in the EPM. Aya in adolescence reduced time on the platform (worse memory) in the MWM. The negative effects observed were not long-lasting because they were not observed in adulthood.	Risk assessment in childhood: P<0.01 Memory in adolescence: P<0.05
Correa-Netto et al. 2017b 55	Male C57BL/6 mice Dose: 1.5 mL/kg (p.o.), 2× week, 12 months Tests: OF, MWM, and EPM	Aya did not cause any change in the weight gain pattern during the 12 months. Aya caused impairment in recall, but not in memory acquisition and habituation in the OF. Aya caused a reduction in locomotion in the OF and EPM, without altering the anxiety state. Aya did not differ from the control in memory acquisition in the MWM. Aya did not impact any variable associated with aging in mice.	Weight gain: P=0.082 Locomotion in the OF: P=0.57 EPM risk assessment: P=0.519; entries into closed arms: P=0.770 MWM memory: P=0.144
Savoldi et al. 2017 56	Adult zebrafish, both sexes Doses: 0.1-3 mL/L, hydric administration Tests: free swimming for 60 min	Higher doses (1-3 mL/L) of Aya caused reduced swimming speed and distance traveled, as well as increased freezing and time at the bottom of the aquarium. Dose of 0.1 mL/L reduced the time at the bottom of the aquarium.	Speed between doses: P=0.0005 Total distance between doses: P=0.0005 Freezing: P<0.05 Time at bottom: P<0.05
da Silva et al. 2019 59	Juvenile marmosets, both sexes Dose: 1.67 mL/300g body weight (*vo*) Protocol: social isolation context (IC) Tests: behaviors, fecal cortisol, and body weight	After IC, typical hypocortisolemia and signs of anhedonia were observed, regardless of sex. Aya reverted cortisol levels to basal levels after 24 h. In males, Aya caused a reduction in scratching and an increase in feeding in the acute phase and after one week. Aya improved body weight in both sexes.	Reduction in cortisol after IC: P<0.03 Reduction in feeding after IC: P<0.01 Increase in self-grooming after IC: P<0.01 Increase in sleepiness after IC: P<0.05 Reduction in scratching and increase in feeding in males after acute dose and one week later: always P<0.05 Improvement in body weight of both sexes after acute dose and one week later: always P<0.01
Andrade et al. 2018 58	Zebrafish embryos Doses: 0-1000 mg/L, hydric form for 96 hpf Tests: Mortality, morphological changes, behavior, and locomotion (sublethal doses)	The lethal concentration of Aya was 236.3 mg/L after 96 h. Aya caused developmental abnormalities, especially at higher doses, such as early or late hatching, loss of balance, edema, and accumulation of red blood cells. A higher dose of Aya caused reduced locomotor capacity.	Early hatching at doses 0.3 and 1.6 mg/L: P<0.05 Late or partial hatching at dose 200 mg/L: P<0.05 in both Loss of balance at dose 40 mg/L: P<0.05 Increased edema and accumulation of red blood cells at dose 200 mg/L: P<0.0520 mg/L reduced locomotor activity at 120 and 144 hpf. For 4 mg/L only 144 hpf. P<0.05, always.
Lobão-Soares et al. 2018 57	Adult zebrafish, both sexes Doses: 0.1 and 0.5 mL/L, hydric administration, acute and chronic (13 days) Test: One-trial Learning task	Acute doses of Aya did not differ from control in the object discrimination index, only chronic doses	Lower discrimination index at chronic doses: P<0.05 Chronic doses with higher average and maximum speeds and distance traveled compared to the control (P<0.05, always)
Nolli et al. 2020 47	Male Wistar rats Protocol: Intermittent access to 2-bottle choice (IA2BC) Doses: Aya 0.5-2× ritual dose (*vo*) and naltrexone (NTX 2 mg/kg, *ip*) Tests: complete blood count, macroscopic evaluation of organs, *c-fos* activation	Aya doses did not reduce Etoh intake. NTX reduced Etoh intake. Absolute brain weight was higher in the unexposed group. NTX indicated a reduction in hemoglobin compared to the Etoh control and an increase in mean corpuscular hemoglobin compared to the group without Etoh exposure. Etoh intake induced c-fos expression in all brain areas analyzed. Aya 0.5× and NTX had lower c-fos expression than Etoh control in the medial orbital cortex (MO). No differences were found in *c-fos* expression between Aya and NTX groups for the other areas of the cortex analyzed	NTX reduces Etoh intake: P<0.05 Brain weight: P<0.05 Hemoglobin: P=0.022 Mean corpuscular hemoglobin: P=0.0074 MO: Aya 0.5x (P<0.01); NTX (P<0.05)
Reis et al. 2020 51	Male Swiss mice Doses: Aya 10 mg/kg 8 days (*vo*); Methylphenidate (Mph) 10 mg/kg (*ip*) Tests: Conditioned place preference (CPP) and c-fos activation in the limbic cortex	There was no preference in the animals during preconditioning. Aya and Mph induced conditioning in the animals. Aya had limited effects while Mph altered c-fos in several limbic areas associated with drug abuse. Aya blocked the effect of Mph on conditioning, restoring functions associated with Mph-induced drug-seeking/desire.	Preconditioning preference: P=0.3388 Conditioning by Aya and Mph: P<0.05 for both Mph with greater c-fos expression in cortical areas compared to preconditioning: P<0.05 Aya blocks the effect of Mph: P<0.05
Colaço et al. 2020 53	Wistar rats, both sexes Doses: Aya 0.5-2× ritual dose; Fluoxetine (Flx, 10 mg/kg) (*vo*), for 28 days Tests: OF, EPM and behaviors, blood count and blood biochemistry evaluation, monoamine dosage, BDNF quantification	Aya was shown to be safe according to clinical, hematological, and macroscopic results (data not shown). No locomotor differences were found between groups in the OF (data not shown).No differences were found in behavioral measures and EPM (data not shown). Aya1 males explored the center less than the OF control and entered the center less than Flx. Aya2 females had higher brain BDNF levels. Aya2 females had higher 5-HT levels, and 5-HIAA levels were lower in Flx. Dopamine and HVA levels did not vary, but DOPAC was higher in females. Noradrenaline was not detected in the samples, and its metabolite MHPG did not vary between groups.	5-HT females: Aya2 *vs* control: P<0.05 5-HIAA females: Flx *vs* control: P<0.05 DOPAC females: Ayas *vs* control: P<0.05 and P<0.01 DOPAC males: Aya2 *vs* control or Flx (both P<0.05) BDNF/brain weight females: Aya2 *vs* control: P<0.05
Xavier et al. 2021 48	Male Wistar rats Dose: 500 mg/kg (*vo*) 15 days (pre-intervention) Intervention: Unpredictable chronic mild stress (UCMS) (30 days) Tests: sweet food intake, adrenal and body weight, biochemical analysis, and OF	UCMS was effective in inducing anhedonia. Aya did not prevent anhedonia-like behavior. Locomotion was not altered by Aya consumption or UCMS-induced stress. Aya counteracted UCMS-induced changes but did not cause changes in unstressed rats.	Reduced intake of sweet foods: UCMS (with or without Aya) *vs* control (P<0.05) Increased adrenal weight/rat hair ratio: UCMS (with or without Aya) *vs* control (P<0.05) Increased TBARS in the cortex: UCMS without Aya vs control (P<0.05)
Gianfratti et al. 2022 52	Male Swiss mice Doses: 0.1-10×, 20× (*ip*) and 0.5-20×, 40× (*vo*); hexobarbital (Hxb) 60 mg/kg (*ip*) Tests: pharmacological screening; acute toxicity; locomotor activity; motor coordination (rota-rod); Hxb-induced sleep time; Conditioned place preference (CPP)	Aya had no effect on motor activity, motor coordination, Hxb-induced sleep latency, or total sleep time. Aya did not cause severe acute toxicity at high doses. Pretreatment with Aya inhibited Etoh-induced CPP and induced CPP when administered alone.	Time in compartment (CPP): Aya+Etoh, P=0.42; Aya, P=0.019; Etoh, P=0.046 Number of entries (CPP): Aya, P<0.05; control x Aya+Etoh, P<0.05; Etoh x Aya+Etoh, P<0.05

All the included studies quantified the substances present in the donated beverages, ensuring the accuracy of the data for comparison with the related literature. Table 2 presents the dosages of each of the active ingredients of interest in Aya and the chromatographic technique used for quantification.

**Table 2:** Dosages of the four active ingredients of interest in ayahuasca tea and chromatographic technique for detection.

Reference	DMT^1^	Harmine	Harmaline	TTH^2^	Chromatography
Alvarenga et al. 2014 46	404 µg/mL	451 µg/mL	124 µg/mL	1482 µg/mL	HPLC-DAD*
Pic-Taylor et al. 2015 49	0.302 mg/kg	3.34 mg/kg	0.261 mg/kg	-	GC-MS/MS**
Oliveira-Lima et al. 2015 50	0.4 mg/100 mg	3.85 mg/100 mg	0.17 mg/100 mg	3.07 mg/100 mg	LC-MS/MS^#^
Correa-Netto et al. 2017a 54	2070 µg/mL	2894 µg/mL	147.5 µg/mL	1893 µg/mL	HPLC-DAD*
Correa-Netto et al. 2017b 55	2070 µg/mL	2894 µg/mL	147.5 µg/mL	1893 µg/mL	HPLC-DAD*
Savoldi et al. 2017 56	0.36 mg/mL	1.86 mg/mL	0.24 mg/mL	1.20 mg/mL	GC-NPD^##^
da Silva et al. 2019 59	0.36 mg/mL	1.86 mg/mL	0.24 mg/mL	1.20 mg/mL	GC-NPD^##^
Andrade et al. 2018 58	0.141 mg/mL	1.56 mg/mL	0.122 mg/mL	-	GC-MS/MS**
Lobão-Soares et al. 2018 57	0.36 mg/mL	1.86 mg/mL	0.24 mg/mL	1.20 mg/mL	GC-NPD^##^
Nolli et al. 2020 47	0.12 mg/mL	1.19 mg/mL	0.08 mg/mL	0.15 mg/mL	LC-MS/MS^#^
Reis et al. 2020 51	0.4 mg/100 mg	3.85 mg/100 mg	0.17 mg/100 mg	3.07 mg/100 mg	LC-MS/MS^#^
Colaço et al. 2020 53	0.26 mg/kg	2.58 mg/kg	0.171 mg/kg	0.33 mg/kg	GC-MS/MS**
Xavier et al. 2021 48	0.28 mg/kg	0.57 mg/kg	0.13mg/kg	0.70 mg/kg	GC-NPD^##^
Gianfratti et al. 2022 52	0.31 mg/mL	0.44 mg/mL	0.75 mg/mL	0.25 mg/mL	GC-NPD^##^

N,N-Dimethyltrypamine (DMT); ^2^Tetrahydroharmine (TTH); *high performance liquid chromatography with diode array detection; **gas chromatogram-tandem mass spectrometry; ^#^liquid chromatogram-tandem mass; ^##^gas chromatography with nitrogen phosphorous detector.

Although the concentration values were converted to a common scale, the quantities of the four active ingredients of Aya used in the studies still vary significantly. This at least in part compromises the interpretation and comparison of the results between the studies. Furthermore, the authors frequently determined the doses to be administered to the animals based on ritualistic doses consumed by individuals weighing 70 kg. Therefore, the consumption indicated in one study differs to some extent from that in another study.

The application of different protocols, resulting in different variables, and the use of different model organisms precluded the feasibility of meta-analysis. Furthermore, the form of exposure also varied between acute and chronic.

Finally, the decision to include studies that assessed biochemical, physiological, and behavioral variables precluded comparisons, other than the limited number of studies and the presence of other conditions.

Table 3 provides a synopsis of the behavioral, biochemical, and physiological changes induced by Aya in the studies included in this review.

**Table 3:** Effects of exposure to ayahuasca (Aya) on behavioral, biochemical, and physiological variables.

Variable	Alteration	Aya dose	Reference
Behavior			
Sex	↓	250-1000 µg/mL	Alvarenga et al. 2014 46
Sex with sleep deprivation	↑	250 µg/mL	Alvarenga et al. 2014 46
	↓	500 µg/mL	Alvarenga et al. 2014 46
Mounts	=	250-1000 µg/mL	Alvarenga et al. 2014 46
Locomotion	↓	15-30 times/ritual	Pic-Taylor et al. 2015 49
	=	1.5 mL/kg 2 times/week	Correa-Netto et al. 2017a 54
	↓	1.5 mL/kg 2 times/week	Correa-Netto et al. 2017b 55
	↓	20 mg/L	Andrade et al. 2018 58
	=	0.5-2 times/ritual	Colaço et al. 2020 53
	=	500 mg/kg	Xavier et al. 2021 48
	=	0.1-40 times/ritual	Gianfratti et al. 2022 52
Motor coordination	=	0.1-40 times/ritual	Gianfratti et al. 2022 52
Balance	↓	40 mg/L	Andrade et al. 2018 58
Hyperlocomotion	↓	300-500 mg/kg	Oliveira-Lima et al. 2015 50
Rearing	↓	15-30 times/ritual	Pic-Taylor et al. 2015 49
Grooming	↓	15-30 times/ritual	Pic-Taylor et al. 2015 49
Swimming	↑	15-30 times/ritual	Pic-Taylor et al. 2015 49
Swimming speed	↓	1-3 mL/L	Savoldi et al. 2017 56
	↑	0.1-0.5 mL/L chronic	Lobão-Soares et al. 2018 57
Distance traveled	↓	1-3 mL/L	Savoldi et al. 2017 56
	↑	0.1-0.5 mL/L chronic	Lobão-Soares et al. 2018 57
Immobility	↓	15-30 times/ritual	Pic-Taylor et al. 2015 49
Freezing	↑	1-3 mL/L	Savoldi et al. 2017 56
Childhood memory	↓	1.5 mL/kg 2 times/week	Correa-Netto et al. 2017a 54
Memory evocation	↓	1.5 mL/kg 2 times/week	Correa-Netto et al. 2017b 55
Memory acquisition	=	1.5 mL/kg 2 times/week	Correa-Netto et al. 2017b 55
Alcohol dependence	↓	300-500 mg/kg	Oliveira-Lima et al. 2015 50
Scratches after social isolation	↓	1.67 mL/300g	da Silva et al. 2019 59
Object discrimination	=	0.1-0.5 mL/L acute	Lobão-Soares et al. 2018 57
	↓	0.1-0.5 mL/L chronic	Lobão-Soares et al. 2018 57
Alcohol intake	=	0.5-2 times/ritual	Nolli et al. 2020 47
Conditioning	↑	10 mg/kg	Reis et al. 2020 51
	↑	0.1-40 times/ritual	Gianfratti et al. 2022 52
Conditioning reversion	↑	10 mg/kg	Reis et al. 2020 51
	↑	0.1-40 times/ritual	Gianfratti et al. 2022 52
Exploration in the OF (male)	↓	1×/ritua	Colaço et al. 2020 53
Anhedonia reversion	=	500 mg/kg	Xavier et al. 2021 48
Biochemistry			
Progesterone	↓	500 µg/mL	Alvarenga et al. 2014 46
Testosterone	=	250-1000 µg/mL	Alvarenga et al. 2014 46
Cortisol	=	250-1000 µg/mL	Alvarenga et al. 2014 46
Cortisol after social isolation	↑	1.67 mL/300 g	da Silva et al. 2019
*c-fos* expression in MO	↓	0.5/ritual	Nolli et al. 2020 47
*c-fos* times by pressure in VO, LO, striatum, and NAc	=	0.5-2 times/ritual	Nolli et al. 2020 47
Block the Mph-conditioning effect	↑	10 mg/kg	Reis et al. 2020 51
BDNF brain (female)	↑	2 times/ritual	Colaço et al. 2020 53
5-HT level (female)	↑	2 times/ritual	Colaço et al. 2020 53
Dopamine level	=	0.5-2 times/ritual	Colaço et al. 2020 53
HVA level	=	0.5-2 times/ritual	Colaço et al. 2020 53
DOPAC level (female)	↑	1-2 times/ritual	Colaço et al. 2020 53
DOPAC level (male)	↑	2 times/ritual	Colaço et al. 2020 53
MHPG level	=	0.5-2 times/ritual	Colaço et al. 2020 53
Physiology			
Ejaculation	=	250-1000 µg/mL	Alvarenga et al. 2014 46
Serotonergic activity	↑	30 times/ritual	Pic-Taylor et al. 2015 49
Alcohol sensitization	↓	300-500 mg/kg	Oliveira-Lima et al. 2015 50
Habituation	=	1.5 mL/kg 2 times/week	Correa-Netto et al. 2017b 55
Body weight	=	1.5 mL/kg 2 times/week	Correa-Netto et al. 2017b 55
Body weight after social isolation	↑	1.67 mL/300g	da Silva et al. 2019 59
Anxiety	=	1.5 mL/kg 2 times/week	Correa-Netto et al. 2017b 55
	↑	1-3 mL/L	Savoldi et al. 2017 56
	↓	0.1 mL/L	Savoldi et al. 2017 56
	=	0.5-2 times/ritual	Colaço et al. 2020 53
Feeding after social isolation	↑	1.67 mL/300g	da Silva et al. 2019 59
Early hatching	↑	0.3-1.6 mg/L	Andrade et al. 2018 58
Late hatching	↑	200 mg/L	Andrade et al. 2018 58
Partial hatching	↑	200 mg/L	Andrade et al. 2018 58
Edemas	↑	200 mg/L	Andrade et al. 2018 58
Red blood cell accumulation	↑	200 mg/L	Andrade et al. 2018 58
Antioxidant activity against induced stress	↑	500 mg/kg	Xavier et al. 2021 48
Induced sleep latency	=	0.1-40×/ritual	Gianfratti et al. 2022 52
Sleep time	=	0.1-40×/ritual	Gianfratti et al. 2022 52

Compared to the control group; ↑: increased effect; ↓: decreased effect; =: no change. OF: open field; MO: medial orbital cortex; VO: ventral orbital cortex; LO: lateral orbital cortex.

### Assessment of study quality

The assessment of each of the ten domains of the SYRCLE tool, along with the final assessment of the risk of bias, is presented in Table 4. The results indicated that, of the total number of studies included, nine were classified as having low risk (64%), three as having moderate risk (22%), and two as having high risk of bias (14%).

**Table 4:** Quality assessment of included studies.

Reference	Domain										Risk of Bias
	1	2	3	4	5	6	7	8	9	10	
Alvarenga et al. 2014 46	L	L	L	L	L	L	L	U	L	L	Moderate
Pic-Taylor et al. 2015 49	L	L	L	L	L	L	L	L	L	L	Low
Oliveira-Lima et al. 2015 50	L	L	L	L	L	L	L	L	L	L	Low
Correa-Netto et al. 2017a 54	L	L	L	L	L	L	L	L	L	L	Low
Correa-Netto et al. 2017b 55	L	L	L	L	L	L	L	H	L	H	High
Savoldi et al. 2017 56	L	L	L	L	L	L	L	L	L	L	Low
da Silva et al. 2019 59	L	L	U	U	L	L	L	L	L	L	Moderate
Andrade et al. 2018 58	L	L	L	L	L	L	L	L	L	L	Low
Lobão-Soares et al. 2018 57	L	L	U	L	L	L	L	L	L	L	Moderate
Nolli et al. 2020 47	L	L	L	L	L	L	L	L	L	L	Low
Reis et al. 2020 51	L	L	L	L	L	L	L	L	L	L	Low
Colaço et al. 2020 53	L	L	L	L	L	L	L	L	H	L	High
Xavier et al. 2021 48	L	L	L	L	L	L	L	L	L	L	Low
Gianfratti et al. 2022 52	L	L	L	L	L	L	L	L	L	L	Low

Domain: 1) Random sequence generation (Selection bias); 2) Baseline characteristics (Selection bias); 3) Allocation concealment (Selection bias); 4) Random housing (Performance bias); 5) Blinding (Performance bias); 6) Random outcome assessment (Detection bias); 7) Blinding (Detection bias); 8) Incomplete outcome data (Attriction bias); 9) Selective outcome reporting (Reporting bias); 10) Other (Other sources of bias). Risk assessment criteria: L: low; U: unclear; H: high.

In the study by Correa-Netto et al. (55), which was assessed as having a high risk of bias, the number of animals in the control group was reduced from nine to four due to deaths during the treatment period. In the Aya group, one animal died, reducing the sample size to twelve individuals. The lost animals were replaced, but the original conditions were not kept, considering the period of chronic exposure. The study by Colaço et al. (53) does not indicate statistical differences between the animals and the control group exposed to the open field, with the exception of males exposed to an intermediate dose of Aya, when locomotor activity was lower.

A number of studies had a moderate risk of bias (46,57,59), mostly because they failed to adequately indicate the inclusion of all animals in the analysis and clearly described the allocation of animals for the proposed experimental conditions.

**Figure 3:**
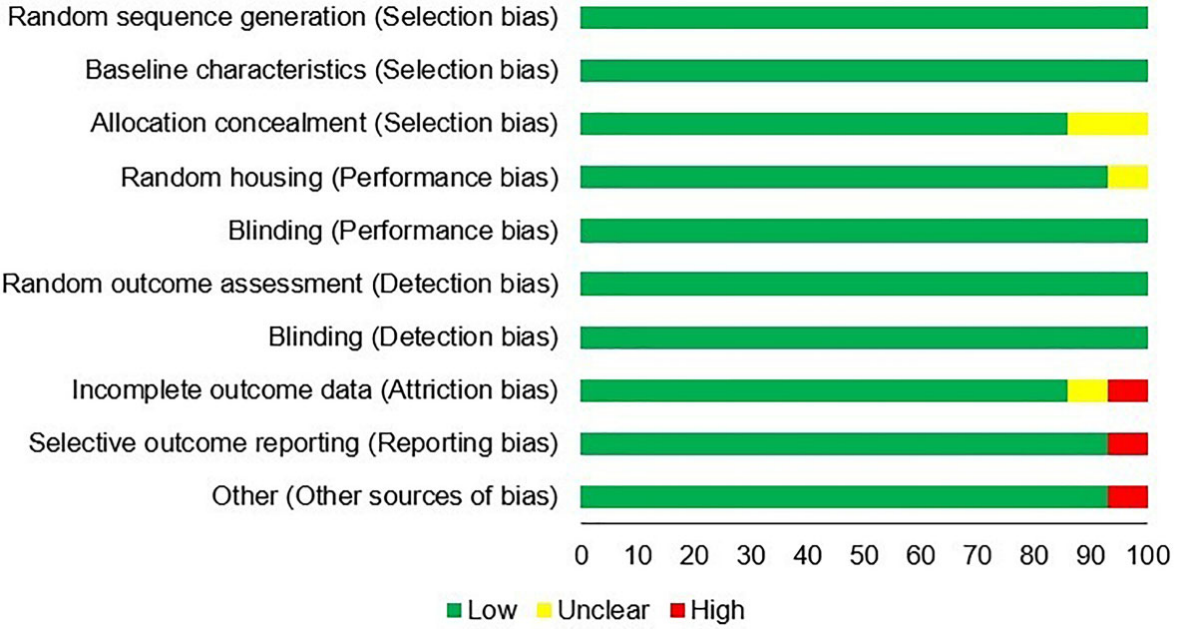
Assessment of the risk of bias (%) of the included studies according to the SYRCLE's RoB tool (adapted from Hooijmans et al. 2014 (45; doi: 10.1186/1471-2288-14-43).

Figure 3 shows the assessment of risk of bias for each of the ten domains of the SYRCLE's Rob tool (45).

## Discussion

In recent decades, the use of Aya has increased significantly, not only its original use in religious traditions, but also its recreational use and, above all, its use as an alternative therapeutic strategy, particularly in cases where conventional treatments prove ineffective (60). A substantial body of human research has documented the effects of Aya, especially its impact on mental state. For instance, Halpern et al. (61) observed that the ingestion of Aya by members of a religious group resulted in enhanced mental clarity and a sense of purpose, in addition to a reduction in anxiety and alcohol abuse. In a further study conducted with Brazilian adolescents, individuals who consumed Aya had a reduction in symptoms of anxiety, body image dysmorphia, and attention deficit compared to those who did not drink the beverage (62).

The therapeutic benefits of Aya consumption in reducing symptoms of anxiety, depression, addiction, and other psychological disorders have been proven (63,64). However, common adverse effects include vomiting, nausea, and diarrhea (65). In addition, auditory and visual hallucinations, as well as motor incoordination, have been reported. Other reported effects include the sensation of being able to fly or even to communicate telepathically with the living or the dead (66,67). Furthermore, there is a plethora of reports describing endocrine, immunological, and cardiovascular changes (68,69), in addition to cellular, neurological, and renal damage (70,71).

Despite the substantial increase in studies on Aya, mostly clinical and observational studies, and the fact that the beverage contains substances that act on diverse metabolic pathways, significant steps in pharmacological bioassays appear to have been suppressed and preclinical studies with animal models, which is the focus of this study, are limited.

A considerable heterogeneity was observed in the variables, animal species, experimental manipulations, substances, and doses of Aya that were used in the included studies. The lack of standardization precludes a reliable comparison of results between studies.

The behavioral effects observed in the studies included were generalized reductions in the activities measured by the researchers, whether motor or associated with learning. These effects may be associated with the combination of the substances contained in the Aya beverage and which ultimately results in increased central serotonergic levels, provoking responses that resemble the negative symptoms of schizophrenia, such as affective blunting and avolition (72). This is attributable to the β-carbolines, harmine, harmaline, and THH, which act as reversible inhibitors of the monoamine oxidase type-A isoenzyme (MAO-A) (63,73), thereby preventing DMT degradation. DMT functions as a 5-HT agonist (-1A, -2A, -2C) and also as agonist of receptors associated with trace amine (potentially TAAR6) (10). This contributes to the elevation of central serotonin levels, which are further enhanced by the fact that THH also acts as a selective serotonin reuptake inhibitor (74). This hypothesis has been observed by Kim (75), who describes that the use of atypical antipsychotics in 5-HT2A significantly improves these negative symptoms.

In addition to the targets described above, DMT has been observed to bind to sigma receptors (Sig-1R). These are chaperone molecules that are also found in the brain and have been shown to mediate diverse signaling pathways, including those associated with oxidative stress (57). Consequently, it can be hypothesized that metabolic pathways associated with combating oxidative stress and inflammation through Sig-1Rbe may be activated due to the presence of DMT and its metabolites (76).

In the clinic, studies have observed that when not inhibited, DMT produces anxiolytic effects due to its agonist action on 5-HT1A (77), thus exerting antidepressant activity. Furthermore, its methoxylated derivative, 5-methoxy-N,N-dimethyltryptamine (5-MeO-DMT), which is also found in plants used in the production of Aya (78), is another serotonergic agonist, but of 5-HT1A and 5-HT2A receptors (79). It has been described as a reducer of locomotor activity and inducer of hyperactivity delay (80-82) and can also be used in depressed patients resistant to usual therapy (83,84).

Biochemical and physiological variables (Table 3) associated with the serotonergic pathway exhibited an increase in animals exposed to Aya (49,53). This phenomenon is likely attributable to the agonistic effect of DMT previously mentioned. However, such effects were not as clearly observed for dopamine and its derivative DOPAC (53). The serotonergic elevation resulting from the combination of DMT and β-carbonyls appears to provide a rational explanation for the observed decrease in ethyl sensitivity (50) and increase in cortisol, body weight, and hunger after social isolation (59). Conversely, exposure to Aya resulted in the disruption of the anticipated hatching sequence of zebrafish eggs (58), thereby underscoring the importance of additional toxicological investigations of the impact of these substances.

Many of the results described in Table 3, regardless of the type of model organism, suggest that animals exposed to Aya experienced blunting and avolition. Such behaviors are characteristic of the negative symptoms of schizophrenia and depression (85,86). Although schizophrenia also has positive symptoms, these were not described or identified in the studies included here. We suggest that the effect of atypical antipsychotics, such as quetiapine, on chronic Aya intoxication in animal models be investigated to see if this psychotropic drug helps to alleviate these symptoms. If successful, Aya could be used to model some of the symptoms of schizophrenia or depression. Some models using drugs to induce depression, such as reserpine (87), are already well established. Stress models that could be used to test this hypothesis include the social defeat stress model (88), mild chronic stress model (89), and learned helplessness model (90).

An important contribution of this review is the identification of gaps in preclinical studies to fully understand the neurochemical, behavioral, and toxicological effects of ayahuasca. In particular, systematic investigations of long-term toxicity and effects on specific physiological systems such as the cardiovascular, renal, and immune systems are lacking.

Given the growing popularity of ayahuasca as a therapeutic tool and the interest in its potential benefits, it is essential that new preclinical studies be conducted with greater methodological rigor. They are essential not only to elucidate the mechanisms of action of ayahuasca, but also to ensure the safety of its use, especially considering the toxic effects on embryonic development and the lack of data on long-term toxicity. Only with a well-defined preclinical basis will it be possible to proceed safely to clinical trials and wider therapeutic use.
